# Pro-malignant properties of STAT3 during chronic inflammation

**DOI:** 10.18632/oncotarget.482

**Published:** 2012-04-20

**Authors:** Marco Demaria, Valeria Poli

**Affiliations:** Buck Institute for Research on Aging, Novato CA, USA; Molecular Biotechnology Center; University of Turin; Turin, Italy

Already in 1863 Rudolph Virchow hypothesized that some classes of irritants, together with tissue injury, enhance cell proliferation and that cancer arises from sites of chronic inflammation. Although now we know that cell proliferation is not a cause of cancer on itself, for the first time his hypothesis suggested a link between cell transformation and inflammation. Many tumors contain activated fibroblasts and macrophages displaying an inflammatory gene expression profile. Interestingly, quantitative aspects of wound repair or inflammatory gene expression often negatively correlate with cancer stage and prognosis: in a sense, tumors act as wounds that fail to heal [1].

Chronic inflammatory states may generate a microenvironment favoring genomic lesions and fostering tumor initiation. The presence of free radicals, such as reactive oxygen intermediates and nitric oxide, leads to oxidative damage and nitration of DNA bases, which in turn increases the risk of mutations. Moreover, the soluble mediators secreted by inflammatory cells such as cytokines and growth factors provide survival and proliferative signals to initiated cells, thereby leading to tumor promotion/progression.

More detailed insights into the role of inflammation in tumor promotion come from several studies involving the transcription factor NF-κB. For example, NF-κB inactivation dramatically decreased tumor size of myeloid cells in a colitis-associated cancer model, reducing the expression of pro-inflammatory cytokines that may serve as tumor growth factors [2].

NF-κB is a ubiquitous transcription factor that regulates genes involved in native and adaptive immune responses. Importantly, NF-κB is often aberrantly activated in human cancers, up-regulating genes involved in the control of survival and proliferation, and is thus considered an important target for drug therapies [3]. A prominent NF-κB target is the gene encoding for the pro-inflammatory cytokine IL-6, which directly affects cancer cells growth and survival through the activation of another transcription factor, the Signal Transducer and Activator of Transcription (STAT) 3. Indeed, chronic inflammation initiates a positive loop between the transcription factor NF-κB, IL-6 and STAT3 that is a highly predisposing condition for cancer, particularly in the colon, the liver and the breast [4].

STAT3 is constitutively activated by phosphorylation on tyrosine in many tumors that often become addicted to its activity [5], and is accordingly often referred to as an oncogene, even though activating mutations are rare. Importantly, STAT3 is prominently constitutively activated at sites of chronic inflammation, where IL-6 levels are invariably high. In an effort to characterize the pro-oncogenic functions of continuous, weak STAT3 activation, we have recently generated knock-in mice expressing physiological levels of the constitutively active STAT3C mutant form [6]. Primary mouse embryonic fibroblasts (MEFs) derived from the STAT3^C/C^ mice show pre-malignant features, such as increased glycolysis, resistance to apoptosis and senescence and accelerated proliferation [7]. When challenged with a second ‘random’ mutation induced through the 3T3 spontaneous immortalization protocol, STAT3^C/C^ MEFs become fully transformed and are able to form tumors in immunocompromised mice [8]. STAT3^C/C^ cells display an accelerated cell cycle, protection from apoptosis and enhanced HIF-1α-dependent aerobic glycolysis. HIF-1α silencing normalizes their glycolysis levels, correlating with decreased cell proliferation and growth, both *in vitro* and *in vivo*.

This finding is of particular relevance for the emerging key role of STAT3 in inflammation-driven cancer. Therefore, in addition to the tumor promotion role described above in coordination with IL-6 and NF-κB, our data suggest that cells exposed to chronic IL-6 signaling, which leads to continuous STAT3 activation like that displayed by the STAT3^C/C^ MEFs, can behave like cells that have undergone a first oncogenic mutation. This first hit provides survival and proliferative signals by inducing pro-proliferative and anti-apoptotic genes and switching cell metabolism towards aerobic glycolysis, believed to sustain the anabolic metabolism required by tumor cells. All these features contribute to a pre-malignant state where a second mutation is sufficient to provide full cell transformation. Interestingly, a state of chronic inflammation with IL-6 accumulation develops with age in mice, primates and humans [9]. This may in turn result in increasingly high chronic STAT3 activation and thus the development of STAT3-dependent tumors. Therapeutic strategies focusing on STAT3 modulation could therefore dramatically decrease the incidence of age-related cancers, lowering the accumulation of the pre-malignant cells pool with aging.
